# Crystal structure and Hirshfeld surface analysis of 2,2′-(phenyl­aza­nedi­yl)bis­(1-phenyl­ethan-1-one)

**DOI:** 10.1107/S2056989022005382

**Published:** 2022-06-07

**Authors:** Farid N. Naghiyev, Victor N. Khrustalev, Marina G. Safronenko, Mehmet Akkurt, Ali N. Khalilov, Ajaya Bhattarai, İbrahim G. Mamedov

**Affiliations:** a Department of Chemistry, Baku State University, Z. Khalilov str. 23, Az, 1148 Baku, Azerbaijan; b Peoples’ Friendship University of Russia (RUDN University), Miklukho-Maklay St.6, Moscow, 117198, Russian Federation; c N. D. Zelinsky Institute of Organic Chemistry RAS, Leninsky Prosp. 47, Moscow, 119991, Russian Federation; d Department of Physics, Faculty of Sciences, Erciyes University, 38039 Kayseri, Turkey; e "Composite Materials" Scientific Research Center, Azerbaijan State Economic University (UNEC), H. Aliyev str. 135, Az 1063, Baku, Azerbaijan; f Department of Chemistry, M.M.A.M.C (Tribhuvan University) Biratnagar, Nepal

**Keywords:** crystal structure, C—H⋯O hydrogen bonds, C—H⋯··π inter­actions, van der Waals inter­actions, Hirshfeld surface

## Abstract

The whole mol­ecule of the title compound, C_22_H_19_NO_2_, is generated by twofold rotational symmetry. The N atom exhibits a trigonal-planar geometry and is located on the twofold rotation axis. In the crystal, mol­ecules are linked by C—H⋯O contacts with *R* ^2^_2_(12) ring motifs, and C—H⋯π inter­actions, resulting in ribbons along the *c-*axis direction. van der Waals inter­actions between these ribbons consolidate the mol­ecular packing. Hirshfeld surface analysis indicates that the greatest contributions to the crystal packing are from H⋯H (45.5%), C⋯H/H⋯C (38.2%) and O⋯H/H⋯O (16.0%) inter­actions.

## Abstract 2

The whole mol­ecule of the title compound is generated by twofold rotational symmetry. In the crystal, mol­ecules are linked by inter­molecular C—H⋯O inter­actions with 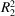 (12) ring motifs, and C—H⋯π inter­actions, resulting in ribbons along the *c-*axis direction. The mol­ecular packing is consolidated by van der Waals inter­actions between these ribbons.

## 1. Chemical context

Functionalized amine and carbonyl compounds are versatile inter­mediates in organic synthesis, material science and medicinal chemistry (Zubkov *et al.*, 2018 ▸; Shikhaliyev *et al.*, 2019 ▸; Viswanathan *et al.*, 2019 ▸; Gurbanov *et al.*, 2020 ▸). *N*,*N*-bis­(phenac­yl)anilines are of particular significance in the fine chemical industry due to their use as precursors of various heterocyclic systems such as piperidine, triazepine, 1,4-di­hydro­pyrazine, 1,4-oxazine, pyrrole and indoles (Zeng & Chen, 2006 ▸; Ravindran *et al.*, 2007 ▸; Paul & Muthusubramanian, 2013 ▸; Yan *et al.*, 2014 ▸). 
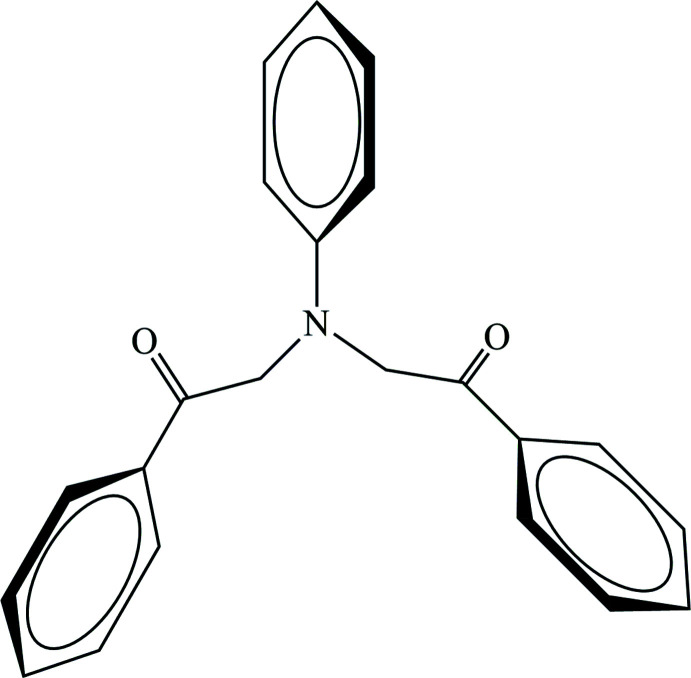


Thus, in the framework of our ongoing structural studies (Naghiyev *et al.*, 2020 ▸, 2021 ▸, 2022 ▸; Khalilov *et al.*, 2022 ▸), we report the crystal structure and Hirshfeld surface analysis of the title compound, 2,2′-(phenyl­aza­nedi­yl)bis­(1-phenyl­ethan-1-one).

## 2. Structural commentary

**Figure 1:**
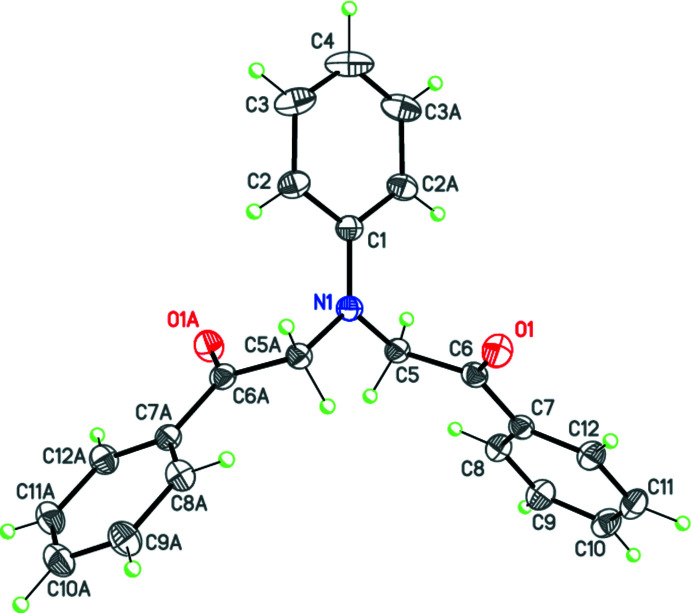
The mol­ecular structure of the title compound. Displacement ellipsoids are drawn at the 50% probability level.

The asymmetric unit of the title compound contains half a mol­ecule, the complete mol­ecule being generated by the twofold rotational axis. Atoms N1, C1 and C4 are located on the twofold rotation axis (Fig. 1 ▸). The N1 atom has a trigonal-planar geometry, and it is bonded to two C atoms (C5 and C5*A*) from two symmetry-related 1-phenyl­ethan-1-one groups and atom C1 of the phenyl ring, which is divided by the twofold rotation axis. The phenyl ring (C1–C4/C2*A*/C3*A*) attached to the N1 atom and the phenyl rings (C7–C12 and C7*A*–C12*A*) of the two symmetry-related 1-phenyl­ethan-1-one groups are oriented at 89.65 (6)° to each other.

## 3. Supra­molecular features and Hirshfeld surface analysis

**Figure 2:**
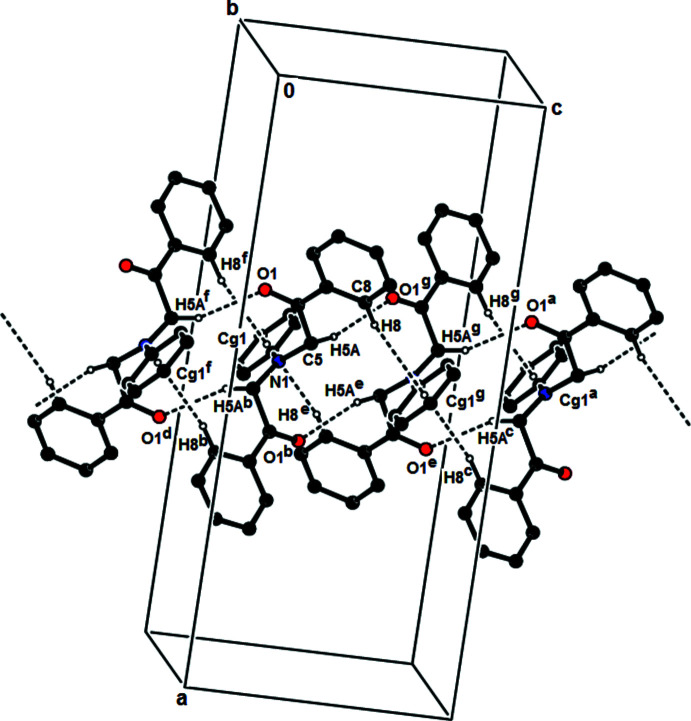
A general view of the inter­molecular C—H⋯O hydrogen bonds, and C—H⋯π inter­actions of the title compound. The hydrogen atoms not involved in the hydrogen bonds have been omitted for clarity. Symmetry codes: (*a*) *x*, *y*, *z* + 1; (*b*) 1 − *x*, *y*, 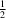 − *z*; (*c*) *x* + 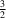 , −*y* + 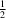 , −*z* + 1; (*d*) 1 − *x*, 1 − *y*, −*z*; (*e*) 1 − *x*, 1 − *y*, 1 − *z*; (*f*) *x*, 1 − *y*, − 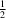 + *z*; (*g*) *x*, 1 − *y*, 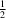 + *z*.

**Figure 3:**
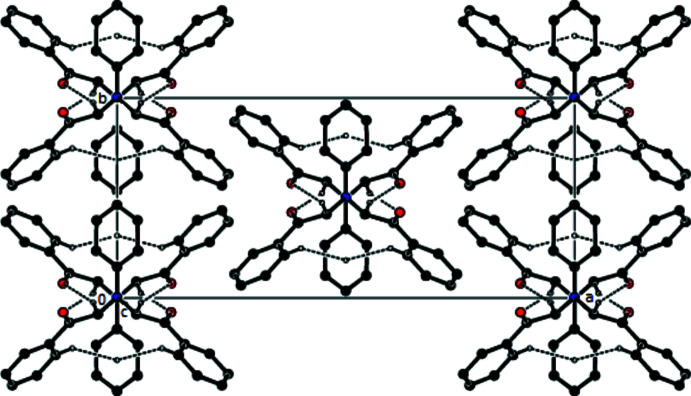
View of the packing down the *c* axis showing C—H⋯O hydrogen bonds and and C—H⋯π inter­actions in the title compound. The hydrogen atoms not involved in the hydrogen bonds have been omitted for clarity.

In the crystal, mol­ecules are linked by inter­molecular C—H⋯O [C5—H5*A*⋯O1(*x*, −*y* + 1, *z* + 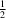 ); 2.51 Å, 158°] inter­actions with 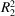 (12) ring motifs, resulting in ribbons along the *c*-axis direction (Bernstein *et al.*, 1995 ▸; Table 1 ▸; Fig. 2 ▸). C—H⋯π inter­actions also contribute to the stronger cohesion of mol­ecules in the ribbons (Table 1 ▸; Fig. 3 ▸). The mol­ecular packing also features van der Waals inter­actions between these ribbons.

**Figure 4:**
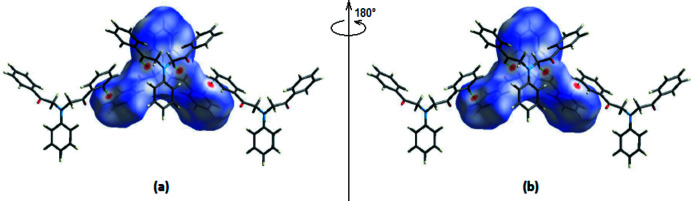
(*a*) Front and (*b*) back sides of the three-dimensional Hirshfeld surface of the title compound mapped over *d*_norm_, with a fixed colour scale of −0.1305 to 1.2546 a.u. The C—H⋯O hydrogen bonds are shown.

*Crystal Explorer*17.5 (Turner *et al.*, 2017 ▸) was used to perform a Hirshfeld surface analysis and generate the associated two-dimensional fingerprint plots, with a standard resolution of the three-dimensional *d*_norm_ surfaces plotted over a fixed colour scale of −0.1305 (red) to 1.2546 (blue) a.u (Fig. 4 ▸). In the Hirshfeld surface mapped over *d*_norm_ (Fig. 4 ▸), the bright-red spots near atoms O1 and H5*A* indicate the short C—H⋯O contacts (Table 1 ▸). Other contacts are equal to or longer than the sum of van der Waals radii.

**Figure 5:**
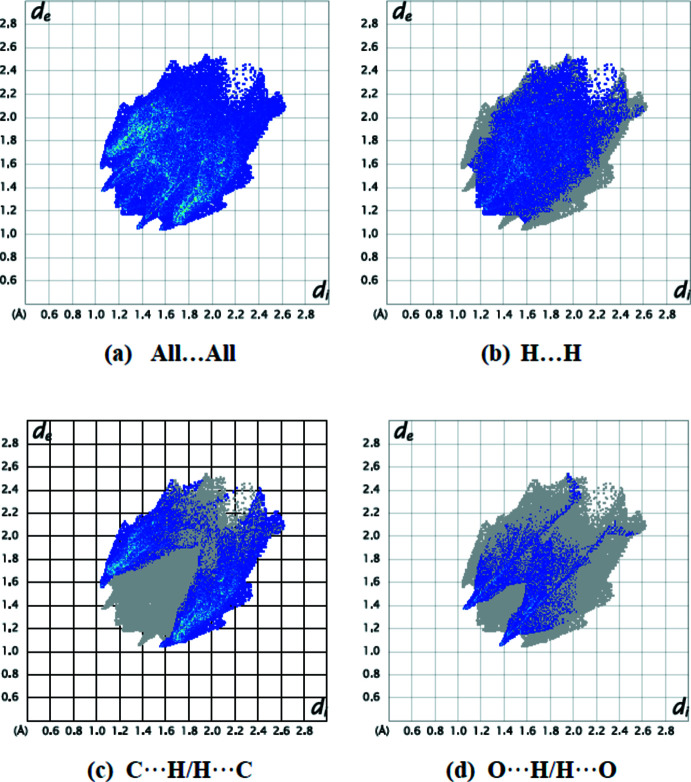
Two-dimensional fingerprint plots of the title compound, showing (*a*) all inter­actions, and delineated into (*b*) H⋯H, (*c*) C⋯H/H⋯C and (*d*) O⋯H/H⋯O inter­actions. [*d*_e_ and *d*_i_ represent the distances from a point on the Hirshfeld surface to the nearest atoms outside (external) and inside (inter­nal) the surface, respectively].

Fingerprint plots (Fig. 5 ▸ *b*–*d*; Table1) reveal that H⋯H (45.5%), C⋯H/H⋯C (38.2%) and O⋯H/H⋯O (16.0%) inter­actions make the greatest contributions to the surface contacts. N⋯H/H⋯N (0.3%) contacts also contribute to the overall crystal packing of the title compound. The Hirshfeld surface analysis confirms the importance of H-atom contacts in establishing the packing. The large number of H⋯H, C⋯H/H⋯C and O⋯H/H⋯O inter­actions suggest that van der Waals inter­actions and hydrogen bonding play the major roles in the crystal packing (Hathwar *et al.*, 2015 ▸).

## 4. Database survey

A search of the Cambridge Structural Database (CSD, Version 5.42, update of September 2021; Groom *et al.*, 2016 ▸) for the *N*,*N*-di­methyl­aniline moiety revealed three structures closely related to the title compound, *viz*. 4-methyl-*N*-[(4-methyl­phen­yl)sulfon­yl]-*N*-phenyl­benzene­sulfonamide [CSD refcode GOBNIW (**I**); Eren *et al.*, 2014 ▸], *N*,*N*′-[(phenyl­imino)diethane-2,1-di­yl]bis­(pyridine-2-carboxamide) [IDIZOM (**II**); Li *et al.*, 2013 ▸] and (2*E*,2′*E*)-dimethyl 2,2′-[(phenyl­aza­nedi­yl)bis­(methyl­ene)]bis­(3-phenyl­acrylate) [XEBWUY (**III**); Sabari *et al.*, 2012 ▸]. Like the title compound, the mol­ecule of (**I**) possesses twofold rotational symmetry. The N atom has a trigonal-planar geometry and is located on the twofold rotation axis. Weak C—H⋯O hydrogen bonds connect the mol­ecules, forming a three-dimensional network. The asymmetric unit of (**II**) contains two independent mol­ecules with similar conformations. In the crystal, N—H⋯O and weak C—H⋯O hydrogen bonds link the mol­ecules into a three-dimensional supra­molecular structure. Weak inter­molecular C—H⋯π inter­actions are also observed. In (**III**), the C=C double bonds adopt an *E* configuration. In the crystal, pairs of C—H⋯O hydrogen bonds link the mol­ecules into inversion dimers.

## 5. Synthesis and crystallization

The title compound was synthesized using the reported procedure (He *et al.*, 2014 ▸), and pale-yellow needle-like crystals were obtained upon slow evaporation from an ethanol/water (4:1) homogeneous solution at room temperature.

## 6. Refinement

Crystal data, data collection and structure refinement details are summarized in Table 2 ▸. All H atoms bound to C atoms were positioned geometrically (C—H = 0.95 and 0.99 Å) and refined using a riding model with *U*_iso_(H) = 1.2*U*_eq_(C). Owing to poor agreement between observed and calculated intensities, eighteen outliers (8 1 3, 1 5 6, 25 0 2, 4 5 3, 2 7 3, 1 2 3, 1 1 6, 7 3 0, 14 3 9, 5 3 0, 4 5 8, 0 4 0, 21 0 2, 7 4 8, 9 10 3, 2 4 0, 23 2 2, 2 8 5) were omitted during the final refinement cycle.

## Supplementary Material

Crystal structure: contains datablock(s) I. DOI: 10.1107/S2056989022005382/tx2050sup1.cif

Structure factors: contains datablock(s) I. DOI: 10.1107/S2056989022005382/tx2050Isup2.hkl

CCDC reference: 2173928

Additional supporting information:  crystallographic information; 3D view; checkCIF report

## Figures and Tables

**Table 1:** Hydrogen-bond geometry (Å, °) *Cg1* is the centroid of the phenyl ring attached to atom N1.

*D*—H⋯*A*	*D*—H	H⋯*A*	*D*⋯*A*	*D*—H⋯*A*
C5—H5*A*⋯O1^i^	0.99	2.51	3.4483 (16)	158
C8—H8⋯*Cg*1^ii^	0.95	2.85	3.6963 (14)	148
C8—H8⋯*Cg*1^iii^	0.95	2.85	3.6963 (14)	148

Symmetry codes: (i) 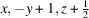 ; (ii) 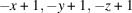 ; (iii) 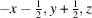 .

**Table 2:** Experimental details

Crystal data	
Chemical formula	C_22_H_19_NO_2_
*M*_r_	329.38
Crystal system, space group	Orthorhombic, *P* *b* *c* *n*
Temperature (K)	100
*a*, *b*, *c* (Å)	20.8269 (2), 9.09843 (10), 9.0158 (1)
*V* (Å^3^)	1708.42 (3)
*Z*	4
Radiation type	Cu *K*α
μ (mm^−1^)	0.65
Crystal size (mm)	0.09 × 0.06 × 0.05

Data collection	
Diffractometer	XtaLAB Synergy, Dualflex, HyPix
Absorption correction	Multi-scan (*CrysAlis PRO*; Rigaku OD, 2021 ▸)
*T*_min_, *T*_max_	0.906, 0.939
No. of measured, independent and observed [*I* > 2σ(*I*)] reflections	21247, 1834, 1746
*R*_int_	0.034
(sin θ/λ)_max_ (Å^−1^)	0.637

Refinement	
*R*[*F* ^2^ > 2σ(*F* ^2^)], *wR*(*F* ^2^), *S*	0.051, 0.142, 1.09
No. of reflections	1834
No. of parameters	115
H-atom treatment	H-atom parameters constrained
Δρ_max_, Δρ_min_ (e Å^−3^)	0.29, −0.23

Computer programs: *CrysAlis PRO* (Rigaku OD, 2021 ▸), *SHELXT2014/5* (Sheldrick, 2015*a* ▸), *SHELXL2018/3* (Sheldrick, 2015*b* ▸), *ORTEP-3 for Windows* (Farrugia, 2012 ▸) and *PLATON* (Spek, 2020 ▸).

## supplementary crystallographic information

### Crystal data

**Table:**

C_22_H_19_NO_2_	*D*_x_ = 1.281 Mg m^−^^3^
*M**_r_* = 329.38	Cu *K*α radiation, λ = 1.54184 Å
Orthorhombic, *P**b**c**n*	Cell parameters from 14002 reflections
*a* = 20.8269 (2) Å	θ = 4.3–79.0°
*b* = 9.09843 (10) Å	µ = 0.65 mm^−^^1^
*c* = 9.0158 (1) Å	*T* = 100 K
*V* = 1708.42 (3) Å^3^	Prism, pale yellow
*Z* = 4	0.09 × 0.06 × 0.05 mm
*F*(000) = 696	

### Data collection

**Table:**

XtaLAB Synergy, Dualflex, HyPix diffractometer	1746 reflections with *I* > 2σ(*I*)
Radiation source: micro-focus sealed X-ray tube	*R*_int_ = 0.034
φ and ω scans	θ_max_ = 79.4°, θ_min_ = 4.3°
Absorption correction: multi-scan (CrysAlisPro; Rigaku OD, 2021)	*h* = −26→26
*T*_min_ = 0.906, *T*_max_ = 0.939	*k* = −11→10
21247 measured reflections	*l* = −10→11
1834 independent reflections	

### Refinement

**Table:**

Refinement on *F*^2^	0 restraints
Least-squares matrix: full	Hydrogen site location: inferred from neighbouring sites
*R*[*F*^2^ > 2σ(*F*^2^)] = 0.051	H-atom parameters constrained
*wR*(*F*^2^) = 0.142	*w* = 1/[σ^2^(*F*_o_^2^) + (0.0811*P*)^2^ + 0.6375*P*] where *P* = (*F*_o_^2^ + 2*F*_c_^2^)/3
*S* = 1.09	(Δ/σ)_max_ < 0.001
1834 reflections	Δρ_max_ = 0.28 e Å^−^^3^
115 parameters	Δρ_min_ = −0.23 e Å^−^^3^

### Special details

**Table:**

Experimental. CrysAlisPro 1.171.41.117a (Rigaku OD, 2021) Empirical absorption correction using spherical harmonics, implemented in SCALE3 ABSPACK scaling algorithm.
Geometry. All esds (except the esd in the dihedral angle between two l.s. planes) are estimated using the full covariance matrix. The cell esds are taken into account individually in the estimation of esds in distances, angles and torsion angles; correlations between esds in cell parameters are only used when they are defined by crystal symmetry. An approximate (isotropic) treatment of cell esds is used for estimating esds involving l.s. planes.

### Fractional atomic coordinates and isotropic or equivalent isotropic displacement parameters (Å^2^)

**Table:**

	*x*	*y*	*z*	*U*_iso_*/*U*_eq_	
O1	0.38204 (5)	0.42787 (12)	0.14622 (11)	0.0346 (3)	
N1	0.500000	0.50356 (17)	0.250000	0.0275 (4)	
C1	0.500000	0.65552 (19)	0.250000	0.0258 (4)	
C2	0.54585 (7)	0.73540 (15)	0.16825 (14)	0.0312 (3)	
H2	0.577660	0.684521	0.112882	0.037*	
C3	0.54488 (9)	0.88824 (18)	0.16798 (17)	0.0432 (4)	
H3	0.575533	0.940593	0.110555	0.052*	
C4	0.500000	0.9651 (2)	0.250000	0.0537 (7)	
H4	0.499999	1.069489	0.250000	0.064*	
C5	0.45804 (6)	0.41853 (14)	0.34384 (14)	0.0256 (3)	
H5A	0.447899	0.476190	0.433961	0.031*	
H5B	0.480651	0.328071	0.375413	0.031*	
C6	0.39556 (6)	0.37610 (14)	0.26663 (14)	0.0262 (3)	
C7	0.35248 (6)	0.26878 (14)	0.34230 (13)	0.0256 (3)	
C8	0.36541 (6)	0.21454 (15)	0.48403 (15)	0.0304 (3)	
H8	0.402012	0.248116	0.537005	0.036*	
C9	0.32461 (7)	0.11135 (17)	0.54737 (16)	0.0362 (4)	
H9	0.333629	0.073789	0.643461	0.043*	
C10	0.27080 (7)	0.06284 (17)	0.47116 (17)	0.0362 (4)	
H10	0.243425	−0.008843	0.514406	0.043*	
C11	0.25697 (7)	0.11905 (17)	0.33172 (16)	0.0353 (4)	
H11	0.219476	0.087802	0.280639	0.042*	
C12	0.29775 (7)	0.22064 (15)	0.26696 (16)	0.0316 (3)	
H12	0.288486	0.257788	0.170838	0.038*	

### Atomic displacement parameters (Å^2^)

**Table:**

	*U*^11^	*U*^22^	*U*^33^	*U*^12^	*U*^13^	*U*^23^
O1	0.0336 (5)	0.0400 (6)	0.0302 (5)	−0.0032 (4)	−0.0024 (4)	0.0071 (4)
N1	0.0260 (7)	0.0226 (7)	0.0338 (8)	0.000	0.0068 (6)	0.000
C1	0.0285 (8)	0.0239 (8)	0.0250 (8)	0.000	−0.0043 (6)	0.000
C2	0.0378 (8)	0.0281 (7)	0.0277 (7)	−0.0037 (5)	−0.0006 (5)	0.0014 (5)
C3	0.0632 (11)	0.0286 (7)	0.0378 (8)	−0.0102 (7)	0.0028 (7)	0.0047 (6)
C4	0.091 (2)	0.0228 (10)	0.0475 (13)	0.000	0.0033 (12)	0.000
C5	0.0251 (6)	0.0241 (6)	0.0277 (6)	−0.0004 (4)	0.0020 (4)	0.0007 (4)
C6	0.0266 (6)	0.0247 (6)	0.0272 (6)	0.0028 (5)	0.0027 (5)	−0.0019 (5)
C7	0.0254 (6)	0.0236 (6)	0.0277 (6)	0.0014 (5)	0.0035 (4)	−0.0028 (4)
C8	0.0292 (6)	0.0326 (7)	0.0292 (6)	−0.0036 (5)	0.0009 (5)	−0.0009 (5)
C9	0.0377 (7)	0.0407 (8)	0.0303 (7)	−0.0064 (6)	0.0040 (6)	0.0041 (6)
C10	0.0352 (7)	0.0367 (7)	0.0366 (7)	−0.0092 (6)	0.0086 (6)	−0.0019 (6)
C11	0.0305 (7)	0.0382 (8)	0.0372 (8)	−0.0086 (6)	0.0014 (5)	−0.0063 (6)
C12	0.0316 (7)	0.0328 (7)	0.0304 (7)	−0.0024 (5)	−0.0009 (5)	−0.0019 (5)

### Geometric parameters (Å, º)

**Table:**

O1—C6	1.2165 (16)	C5—H5B	0.9900
N1—C1	1.383 (2)	C6—C7	1.4913 (18)
N1—C5	1.4415 (14)	C7—C8	1.3960 (19)
N1—C5^i^	1.4416 (14)	C7—C12	1.3973 (19)
C1—C2^i^	1.4082 (16)	C8—C9	1.3892 (19)
C1—C2	1.4082 (16)	C8—H8	0.9500
C2—C3	1.391 (2)	C9—C10	1.387 (2)
C2—H2	0.9500	C9—H9	0.9500
C3—C4	1.382 (2)	C10—C11	1.388 (2)
C3—H3	0.9500	C10—H10	0.9500
C4—H4	0.9500	C11—C12	1.384 (2)
C5—C6	1.5254 (17)	C11—H11	0.9500
C5—H5A	0.9900	C12—H12	0.9500
			
C1—N1—C5	122.46 (7)	O1—C6—C7	121.50 (12)
C1—N1—C5^i^	122.46 (7)	O1—C6—C5	120.45 (11)
C5—N1—C5^i^	115.08 (14)	C7—C6—C5	118.05 (11)
N1—C1—C2^i^	121.07 (9)	C8—C7—C12	119.44 (12)
N1—C1—C2	121.07 (9)	C8—C7—C6	122.28 (12)
C2^i^—C1—C2	117.86 (17)	C12—C7—C6	118.27 (12)
C3—C2—C1	120.47 (14)	C9—C8—C7	119.81 (13)
C3—C2—H2	119.8	C9—C8—H8	120.1
C1—C2—H2	119.8	C7—C8—H8	120.1
C4—C3—C2	120.98 (15)	C10—C9—C8	120.38 (13)
C4—C3—H3	119.5	C10—C9—H9	119.8
C2—C3—H3	119.5	C8—C9—H9	119.8
C3^i^—C4—C3	119.2 (2)	C9—C10—C11	119.97 (13)
C3^i^—C4—H4	120.4	C9—C10—H10	120.0
C3—C4—H4	120.4	C11—C10—H10	120.0
N1—C5—C6	112.65 (9)	C12—C11—C10	120.05 (13)
N1—C5—H5A	109.1	C12—C11—H11	120.0
C6—C5—H5A	109.1	C10—C11—H11	120.0
N1—C5—H5B	109.1	C11—C12—C7	120.32 (13)
C6—C5—H5B	109.1	C11—C12—H12	119.8
H5A—C5—H5B	107.8	C7—C12—H12	119.8
			
C5—N1—C1—C2^i^	−6.40 (9)	O1—C6—C7—C8	−176.33 (12)
C5^i^—N1—C1—C2^i^	173.60 (9)	C5—C6—C7—C8	4.37 (18)
C5—N1—C1—C2	173.59 (9)	O1—C6—C7—C12	4.30 (19)
C5^i^—N1—C1—C2	−6.41 (9)	C5—C6—C7—C12	−175.00 (11)
N1—C1—C2—C3	179.32 (10)	C12—C7—C8—C9	1.3 (2)
C2^i^—C1—C2—C3	−0.68 (10)	C6—C7—C8—C9	−178.06 (12)
C1—C2—C3—C4	1.4 (2)	C7—C8—C9—C10	−0.6 (2)
C2—C3—C4—C3^i^	−0.69 (11)	C8—C9—C10—C11	−0.9 (2)
C1—N1—C5—C6	93.41 (9)	C9—C10—C11—C12	1.7 (2)
C5^i^—N1—C5—C6	−86.59 (9)	C10—C11—C12—C7	−1.0 (2)
N1—C5—C6—O1	−8.70 (17)	C8—C7—C12—C11	−0.6 (2)
N1—C5—C6—C7	170.61 (11)	C6—C7—C12—C11	178.84 (12)

Symmetry code: (i) −*x*+1, *y*, −*z*+1/2.

### Hydrogen-bond geometry (Å, º)

*Cg1* is the centroid of the phenyl ring attached to atom N1.

**Table:**

*D*—H···*A*	*D*—H	H···*A*	*D*···*A*	*D*—H···*A*
C5—H5*A*···O1^ii^	0.99	2.51	3.4483 (16)	158
C8—H8···*Cg*1^iii^	0.95	2.85	3.6963 (14)	148
C8—H8···*Cg*1^iv^	0.95	2.85	3.6963 (14)	148

Symmetry codes: (ii) *x*, −*y*+1, *z*+1/2; (iii) −*x*+1, −*y*+1, −*z*+1; (iv) −*x*−1/2, *y*+1/2, *z*.
